# Foreign Summary

**Published:** 1839-08-31

**Authors:** 


### FOREIGN SUMMARY.

*Clinical Lecture on Porrigo Favosa,—Eczema,—Impetigo.* By Robert Carswell, M. D.—Gentlemen: We have had, and still have in our wards, several interesting cases of cutaneous diseases, viz., two cases of porrigo favosa; one case of impetigo; two cases of eczema; and one case of psoriasis guttata.

I shall, in the present lecture, treat more at length of some of the cases than of others, and chiefly with the view of impressing you with the importance of an accurate knowledge of the diagnostic characters of cutaneous diseases. I have, already, on a former occasion, endeavoured to point out to you the necessity and importance of studying these diseases practically,—of investigating them for yourselves,—of making yourselves familiar with their *elementary* physical or anatomical characters; and for these obvious reasons, viz., that the classification of cutaneous diseases now almost universally adopted—that of Willan and Bateman—is founded on these elementary characters; that it is, therefore, only by means of an accurate knowledge of them that you can recognise the individual existence of any special disease of the skin, and distinguish it from any analogous but different disease of this tissue. That it is also in consequence of your being in possession of an accurate knowledge of these elementary characters, that you can turn to your own advantage, and that of your patients, the opportunities you now possess, and which you must, at some future period, almost daily meet with in practice. And I may again repeat, that it is by means of this knowledge alone that you can avail yourselves of the assistance to be derived from the recorded experience of those who have studied cutaneous diseases in an especial manner, and who ought to be your guides in the successful treatment of them.

I insist the more on this part of our subject, that so little attention is paid to cutaneous diseases by medical men at that period of their studies when they can best acquire a knowledge of them; that is to say, when students, and when attending hospital practice, where there are always to be seen a certain number and variety of these diseases. It is true that they are small in number, compared with other diseases which, from their urgency, severity, and danger, require in-door hospital relief, and the employment of remedial means which cannot otherwise be obtained. Still there are a great many cutaneous diseases, independently of those accompanied with fever, which render the subjects of them unfit for the discharge of their daily labours; which occasion much annoyance, and even suffering, for months or years; which undermine the general health; which disfigure various parts of the body; which spread by contagion; or which place the sufferers in a state to be loathed and avoided. Few, comparatively speaking, of such cases, are received into hospitals. A much greater number of this class of patients apply for relief as out-patients, or go to dispensaries; and a still greater number there must be who cannot take advantage even of the limited means possessed by most of the charitable institutions in London, for the successful treatment of cutaneous diseases.

I notice this latter circumstance that I may take advantage of the present opportunity of expressing my opinion as to the importance and necessity of having in this vast metropolis, either separate establishments, or in connection with hospitals, for the treatment of cutaneous diseases. Without such establishments our knowledge will remain, as hitherto, inaccurate and incomplete, and the treatment of them desultory, empirical, and unsuccessful.

In every science of observation, the accuracy and extent of the knowledge which we acquire of the objects which it embraces, are in proportion to the extent and frequency of the opportunities we possess of seeing and comparing these objects; and this becomes the more necessary the greater their number and diversity, as is the case with the diseases of the skin. Such opportunities and advantages can, I repeat, only be obtained by bringing together a great many of these diseases, and which would speedily be accomplished were they to receive that special attention from physicians to which they are entitled.

Of the five cases of diseases of the skin to which I propose to direct your attention to-day, there are, as I have said, two of porrigo favosa, the only two cases of this kind we have had in my wards during the last six months. One of the cases was an example of the worst form of porrigo favosa; that is to say, of great extent and of long duration. The other was more limited, and of much shorter duration. There was no difficulty in recognising the physical characters of the disease in either, although they were much more marked in the former than in the latter, both as to the cup-shaped appearance and colour of the pustules, and the patches formed by their extension and subsequent union. But as some of you may not be aware of what constitutes the elementary character of porrigo favosa, I may repeat what I stated on a former occasion, that this contagious pustular affection of the scalp consists in the presence of what is called the *favous pustule*. This pustule is formed by the deposition of a minute quantity of pus, which concretes almost immediately into a pale yellow or straw-coloured substance, having a defined circular edge, hardly, if at all, rising above the surface of the skin, and surrounded by a slight blush of red. The successive effusion and concretion of the matter proceeds from the centre towards the circumference, in which direction it accumulates, and thereby raising the circular edge of the crust, and giving to it that *cup-shaped* appearance by which it is recognised. The size of these crusts, or concretions, varies from one to two lines, to half or three-quarters of an inch in diameter. They are distinct at the commencement, but become confluent during their progress, and are sometimes confounded together into a large, dry, brittle mass, resembling a mixture of sulphur and plaster. Even in this state, however, of agglomeration, traces of the primitive or elementary character of the disease are perceptible, viz., numerous round or irregular depressions, indicating the situation and number of the original favi. The most of these appearances you will recognise in the description given of the following case of porrigo favosa.

William Tobin, æt. 18, was admitted on the 6th of April. He is of a scrofulous habit and sanguineous temperament, and has enjoyed very good health, with the exception of headach, induced by the eruption on the scalp, which first appeared eight years ago, and has continued up to the present time, gradually getting worse, being attended with considerable itching and smarting, especially in warm weather.

On admission, the head was the seat of an extensive eruption, occupying the upper, anterior, and posterior parts, and the patient stated that at one time it extended over the eyebrows. On all these parts the eruption does not present the same characters. On the upper, anterior, and lateral parts of the head, towards the temples, it presents the characters of porrigo favosa; over the occiput, those which bear a resemblance to the granular form of impetigo. On this part the hair is plentiful, but short and crisp, whereas on the former parts few traces of it remain. The most advanced part of the favous eruption presents patches, distinct from each other, five in number, and from one to two inches in diameter, of an irregular, but somewhat circular, figure. The margin of each patch is uneven, thick, and elevated; its surface rough, dry, and fissured, of a yellowish-white colour, very much resembling a mixture of sulphur and mortar, more elevated in some parts than in others; of a loose texture, and in many places cellular, assuming the appearance of honeycomb. Besides these large patches, constituting the advanced form of the disease, and the result of the accumulation of the concrete secretion, and agglomeration of the original pustules, these latter are seen in a few situations, presenting their elementary characters; that is to say, they appear in the form of small, flat, straw-coloured pustules, quite distinct, with an elevated border, depressed centre, and slightly inflamed base. The intervening skin over a great part of the scalp is red, in several parts greatly congested, and traversed by numerous varicose vessels. There is considerable itchiness, but no great heat. Numerous large pediculi burrow in the fissures and sulci of the larger patches, and among the roots of the hair, on the posterior part of the head. The whole surface exhales an offensive and nauseous odour.

The lymphatic glands of the neck and left axilla, are greatly enlarged and very hard. The general health is pretty good; appetite very good; tongue clean; bowels regular; urine not albuminous; impulse of heart too strong and too extended.

From the remarks I have made on the elementary characters of this form of pustular eruption of the scalp, you will readily recognise in the description of this case, the porrigo favosa. There is no other pustular eruption with which it could be confounded, except the porrigo *scutulata;* and here the mistake would be of little importance, as it is a disease of the same nature, similar in its elementary characters, arising, like the former, in the favous pustule,—communicable, like it, by contagion, and requiring the same means of treatment. The porrigo favosa is a disease which, when once seen, can hardly ever be confounded with any of the other and more common non-contagious pustular eruptions of the scalp; all its physical characters are so peculiarly well marked, and leave a defined and lasting impression on the mind. Those of you who have not seen this case when the patient was admitted, and before the incrustations were removed, may now obtain an accurate idea of the original appearance of the disease, so faithfully represented by Mr. Tuson in the wax model before you.

The extent of the disease in this case; the great length of time which it had existed, and especially the extremely unhealthy condition of the scalp in general, were circumstances which combined to render the cure both tedious and difficult. It is easy to remove the incrustations, however extensive, by repeated poulticing; but the morbid condition of the tissue of the scalp, and of the bulbs of the hair, often resists, for a long period, the most assiduous and judicious use of remedies. It is not so much the reproduction of the original disease that retards the cure, as the repeated production of other pustular and vesiculo-pustular eruptions, the consequence of the diseased state of the skin. It is this latter, therefore, which we have chiefly to combat in bad cases of porrigo favosa, and to which, in this case, after the removal of the incrustations, the principal part of the treatment has been directed. After the application of poultices for two days, the scalp was completely cleansed of the incrustations. The hair was clipped short, and for some time the poultices were continued during the night, and the water-dressing applied during the day. Under this simple antiphlogistic treatment, considerable improvement followed, and was afterwards farther advanced by the occasional application of leeches to the most inflamed and congested parts of the scalp. With the same view, and also to diminish the tension and turgescence of the scalp, and effect the obliteration of the vessels with which it was so thoroughly penetrated, scarification, by means of a fine scalpel, was had recourse to, but could not be persevered in to any extent, in consequence of the great pain complained of by the patient. I expected to have derived great advantage from this method of treatment, which I have found extremely successful in chronic cases of circumscribed impetigo of this part of the body, accompanied by great thickening and vascularity of the scalp. It removes the tension of the affected part, diminishes the swelling, empties the enlarged vessels, and affords a free issue to the puriform secretion, which is either infiltrated, or exists in the form of numerous small abscesses.

During the early progress of the case, copious eruptions of acores pustules took place from time to time. This was occasioned chiefly by the use of the linseed poultices, and which always happens when not made of the fresh material. This occurrence, however, was obviated by using the potato-poultice instead, the pustules which now make their appearance from time to time being comparatively few in number. During the last two weeks or more, these poultices have been applied at night, and a lotion of the subacetate of lead, or the simple water-dressing, during the day. Leeches also, from eight to ten in number, have been applied several times to the most inflamed parts, during this period.

The improvement which has been effected by these antiphlogistic means has been very considerable; but there is yet much to be done to accomplish a cure of the disease. Although the tumefaction and vascular turgescence of the scalp have nearly disappeared, there is still much redness and tension, which will require the same means of treatment for some time to come. When these morbid conditions have been still further reduced, and should we retain this patient a sufficient length of time in the hospital, as an in-patient, we may then employ, with advantage, various stimulating, or otherwise modifying agents, such as alkaline and sulphureous lotions, the nitrate of silver, and other remedies of this kind, which experience has shown to be useful in the last stage of severe cases of porrigo favosa.

In the treatment of this case, we have not had recourse to the removal of the hair, recommended by several dermatologists, as a necessary part of the treatment of this disease. I am not disposed to attach much importance to this practice. At all events, the removal of the hair may be accomplished much more easily and speedily by the use of the alkaline ointment, or lotion, recommended by Biett, than by any of the means formerly employed. The ointment may consist of from one to two drachms of the sub-carbonate of potash, or of soda, to an ounce of hog's lard; and the lotion of the same quantity of these salts to a pint of water.

I may remark, that the parts which were covered by the incrustations remain perfectly bald; the skin is quite smooth and glossy, and presents a somewhat reticulated aspect. Owing to the long duration and severity of the disease, there is no probability that the hair will ever be reproduced. In milder cases this sometimes happens, the new hair, however, having a somewhat woolly and crisp appearance.

No general treatment has been employed in this case, further than attention to the state of the bowels, and the use of the iodide of potassium, which was indicated from the scrofulous enlargement of the lymphatic glands of the neck. It is doubtful whether any favourable change has followed its administration. If these glands are less hard than formerly, they are certainly not diminished in bulk.

I may say a few words on the supposed causes of porrigo favosa. It is, in itself, contagious, communicable by contact; but it occurs in many instances independently of this source, under a variety of circumstances, as to age, sex, and constitution. Biett states that its development is excited under a variety of circumstances, such as a deficiency of the necessary articles of food; misery and filth; living in unhealthy, ill-aired, damp situations. That it occurs chiefly in persons of a soft, lymphatic, and highly scrofulous constitution, although it may be met with in young persons presenting all the attributes of health and strength. Under such circumstances, it is obvious that we are, as yet, ignorant of the real cause of this disease, as well as of that state of the constitution which renders individuals susceptible of its contagious influence; for it is not always transmissible by contact, as has been proved by experiment; and many boys in the same school, equally exposed with those who contract it, escape altogether free from it.

The second case of porrigo favosa is a good example of the disease, occurring in an otherwise healthy child, of five years of age. We have not been able to obtain a full history of the case. The father is said to have had a scorbutic eruption on the face. A brother and sister have had eruptions on the head, but the precise nature of them has not been ascertained.

The eruption in this child had existed nearly five months, and he was treated for some time as an out-patient. When admitted into our wards, a number of favous pustules, and patches of incrustations, of a pale, yellowish-gray colour, of a brittle consistence, the largest not more than the fourth of an inch in diameter, occupied the upper and posterior parts chiefly of the scalp. They were accompanied by very little redness of the surrounding skin; no exudation, or other morbid appearance of the rest of the head. The general health was good; there was no enlargement of the abdomen, no derangement of the digestive organs; appetite good; sleep natural.

The elementary characters of the porrigo favosa were readily recognised in this case, although not so marked as in the former one. Several of the pustules presented the depressed centre and elevated border; and the incrustations the dry plastery look and colour peculiar to them.

The favourable state of the general health, the limited extent of the disease, and the absence of inflammation of the scalp, except in the immediate vicinity of the patches, were circumstances which augured a speedy, and, probably, permanent cure of the disease. The incrustations were removed by poultices, and the head cleansed by frequent ablution with warm water and soap. The alkaline ointment was afterwards used for some time to effect the removal of the decayed hair, and also to stimulate the less inflamed parts of the diseased cutis. And, lastly, the nitrate of silver was applied to those points where fresh pustules appeared, or where the skin was excoriated. No trace of the eruption remained at the end of six weeks, except complete baldness of the parts which the former had occupied. Full diet was allowed, and the bowels were regulated by occasional small doses of calomel and rhubarb.

The success which followed the use of the nitrate of silver in this case, was partly attributable to the circumstance of the disease having ceased to spread, although it certainly also arrested the development of several pustules which appeared during the progress of the treatment.

The next case is one of impetigo, which occurred in a boy ten years of age, of sanguineous temperament. He had generally enjoyed good health, although he has had two or three times an eruption on the head. The first occurrence of the eruption was two years ago, just after the death of his father, who, he said, had an eruption on the head at the time of his death, and from whom he supposed he caught it, although a brother and sister, equally exposed with himself, had no eruption.

The present eruption occurred two months ago, a few pustules only occupying a small spot, which discharged a good deal, and gradually spread.

On his admission there was an eruption on the top of the head, on the left side, consisting chiefly of incrustations, of a dry, rather brittle appearance, of a dirty, yellowish-gray, or brownish colour, intermixed with, and spreading amongst, the hair. The elementary character of the eruption was not seen on any part of the head at this time, but it soon after appeared in the form of the acores pustule; that is to say, a small, prominent pustule, larger than the psydraceous, containing a yellow-coloured fluid pus, which, after a short period, burst, and spread its contents amongst the hair, which became converted into yellowish-gray or brown crusts. By means of these characters we recognised the presence of impetigo. The prominent pustule, with its fluid contents, were sufficient to establish the diagnosis, and to distinguish the eruption from porrigo favosa and porrigo scutulata, the two contagious forms of pustular eruptions of the scalp. When we can accomplish this important point, we need not trouble ourselves to find out whether an eruption of the scalp is one of porrigo larvalis, or porrigo granulata, as they have improperly been called, as both of these eruptions are modified forms of impetigo, or of eczema impetiginodes, and are not propagated by contagion.

The general health of this boy was good, and remained so while he was under treatment, except on one occasion, when he had fever for two days, from derangement of the bowels. The treatment continued for about the same length of time as in the preceding case. It was commenced by the removal of the hair, and the application of poultices. The patient was allowed full diet, and the bowels were regulated by small doses of calomel and rhubarb every alternate night. The removal of the hair on the affected parts was accomplished by the daily use of the alkaline ointment, and washing with warm water and soap.

On several occasions, however, the eruption spread, or appeared on previously healthy parts of the head, and also on the ears, by the formation of pustules, to which the same means of treatment was applied. After this had ceased to occur, and the inflammatory stage had subsided, the zinc ointment was employed for some time; and, lastly, the nitrate of silver, which completely checked the further progress of the disease.

The affected parts of the head did not present that baldness and glossy appearance of the skin, which were observed in the cases of porrigo favosa. On the contrary, the hair was only thinned and shortened, and the skin presented numerous dark points, indicating the protrusion of others from its surface. In most cases of impetigo, which constitute a great number of the pustular eruptions of the scalp, the treatment employed in this case will prove successful, when the general health is not impaired, and proper attention is paid to food, clothing, and exercise. In debilitated and scrofulous children, however, general treatment requires special attention. Besides attending to the circumstances just mentioned, and regulating the biliary and intestinal secretions, the warm or shower-bath, according to circumstances, must be employed, combined with mild tonics, together with the use of those remedies which act in a special manner on the capillary circulation, and thereby promote the important functions of secretion and absorption. And no remedy in our possession exercises such a speedy and beneficial operation in this respect as the iodide of iron, particularly when aided by the other general means to which I have alluded. In debilitated, scrofulous, and especially anemiated children, its remedial agency is often remarkable. I need not remind you that its use is counter-indicated in all cases of gastro-intestinal irritation, and even where local inflammation of a sthenic character is present. These states must first be subdued by proper antiphlogistic treatment.

The next case which I have to lay before you is that of Phœbe Avery, aged 48, admitted the 14th of May, affected with eczema. The following is the history of her case:—

She is of rather tall stature, stout conformation, and sanguineous temperament. She is a housekeeper; married; and has had ten children, four of whom are living. Her habits and mode of life are regular. She is a native of Hampshire, but has lived in town for the last twenty-six years, in an open and dry place. She had small-pox at the age of eleven, and scarlet fever, rather severely, at the age of twenty-two. She had influenza five years ago, which left her in a weak state. About five or six weeks before Christmas, she received an injury from a carriage wheel falling upon her leg, bruising the skin, and otherwise injuring the limb, to some extent. A linseed poultice was employed, and the wound healed by Christmas; but weakness and tenderness of the limb remained, with redness, and itching of the ankle, which gradually increased, when she applied for medical advice, and was ordered to employ cooling lotions, which she says served only to aggravate the symptoms. Three weeks ago she complained of aching pain in the knee, for which she had a liniment. This brought out a vesicular eruption on the knee, ankle, and shin-bone. She next applied a stimulating ointment to the leg, the effect of which was a rapid extension and coalition of the vesicular eruption; and within the last ten days, small pimples have appeared on various parts of the body. On Friday last she was admitted under Mr. Liston's care; on the 14th (May) transferred to our wards.

*Present Symptoms.*—Extensive redness and severe itching of the right leg, the redness assuming in some parts a purplish colour, thickly set with vesicles, in various stages of development. There is also a quantity of scales, the result of the concretion of the fluid discharged from the ruptured vesicles. The limb is considerably swollen, from œdema, tense, and hard, particularly around the ankle-joint, and very painful on pressure. The eruption is also observed, in a slight degree, on the chest, arms, thighs, &c., apparently dying away. Appetite not impaired; tongue slightly coated; bowels irregular; pulse strong, full, and somewhat accelerated; urine scanty, and rather high coloured; catamenia have not appeared for the last six weeks.

This case is interesting, not so much from the cause in which the eczema originated, viz., a mechanical injury, as from the lesson it affords, by the subsequent aggravation and extension of the disease, from improper treatment. Cold lotions were applied at the commencement to the limb; and although the patient thought they did harm, they would, most likely, had they been longer employed, conjointly with rest, have effected a cure in this, the early stage of the disease. It is, however, obvious, that the stimulating ointment, of whatever nature it may have been, was highly injurious, as great increase and extension of the vesicular eruption, and, consequently, of the inflammation, immediately followed its use. Indeed, it does not appear, from the history of the case, that the disease was eczema at the commencement. It appears rather to have been erythema, or slight erysipelas, and was converted into eczema by the injudicious employment of heating and stimulating ointments; and which are always injurious in this stage of the disease, and more especially when the inflammation occupies the entire substance of the cutis, and, at the same time, a large extent of surface.

When the case came under our observation, the elementary character of the eruption was well marked, viz., numerous vesicles scattered over a highly inflamed surface, accompanied by a serous exudation, some of which had become dried into thin, laminated scurf. These appearances served at once to establish the diagnosis. It differed from erythema and erysipelas by the presence of the small vesicles, and would have been distinguished from psoriasis inveterata, which is often accompanied by much redness, even had the vesicles been absent, by the serous exudation. We had, therefore, no doubt on this head, nor as to the means of treatment to be employed. As the pulse was full and strong, as there was a considerable degree of abdominal congestion, perhaps partly dependent upon the period of life at which the catamenia are about to cease, venesection was ordered to twelve ounces; a draught, composed of two drachms of the sulphate of magnesia, fifteen minims of dilute sulphuric acid, in an ounce and a half of water, three times daily; and a lotion of the subacetate of lead to be applied constantly to the leg; the patient to remain quiet in bed, and to be kept on low diet.

The heat, irritation, and itching of the limb were immediately and considerably relieved by the bleeding. Four days after, it is stated, that the local symptoms are much improved; the swelling and redness are diminished; the skin is less tense; the vesicles on the general surface have degenerated into scurfy films, which are now being shed. The bowels are regular; the tongue clean; appetite good. She was now put on middle diet; and, as the bowels had been very freely acted upon by the mixture, the sulphate of magnesia was reduced to one drachm. Some days after the mixture was altogether omitted, from its having occasioned purging, followed by some weakness; and, as the patient complained of much acidity and some thirst, she was ordered a draught, composed of ten minims of the liquor potassæ, and four minims of the dilute hydrocyanic acid, in ten drachms of water; to be taken three times in the course of the day.

The local, cooling, and sedative treatment was continued up to the 28th, that is to say, for two weeks; but the improvement had not advanced much for some days previously. This was now the period for adopting with success the stimulating plan of treatment; the swelling and tension had considerably diminished; the heat and itching were also but slight; and the redness had assumed, over the greater part of the leg, a purple, or, I may say, a cold-looking colour. I, therefore, decided on using a solution of the nitrate of silver, of the strength of fifteen grains to the ounce of distilled water. This was applied to the upper part of the leg, including about three inches of the skin in breadth all round, and to be used in the same manner, after an interval of two or three days, to the same extent of surface each time, on the remainder of the leg.

Four days after the first application, the report says that the leg is much softer to the touch, and the redness disappears much more readily on pressure. Four days after, the 4th of June, it is stated that the local symptoms are all much relieved where the solution has been applied; on the 8th, that the leg is softer and less tense, and begins to assume the natural appearance, as far down as the last application of the nitrate of silver; and on the 11th, that it is in all respects better. The solution was continued with the same favourable result over the whole of the leg. Last week the patient had walked about the ward, too soon, it would appear, for the leg was somewhat swollen on the following day, and accompanied by an increase of the redness, especially about the ankle and foot. This has been subdued by the application of the lotion, and a bandage is to be worn from the toes up to the knees, to prevent a similar occurrence, and to afford a support to the limb, which is still weak. After omitting the saline purgative, the bowels were kept free by the occasional use of the compound extract of colocynth and blue pill. The diet of the patient was gradually increased from low to full diet. She may now be considered convalescent, and may probably leave the hospital in the course of a week.

The second case of eczema is one of considerable severity from its extent, and the length of time it has existed. The patient, Isaac Lansdowne, 49 years of age, was admitted the 6th of this month, (June.) He is of a florid complexion, slight conformation, with distortion of the lower extremities; a porter, single, and of intemperate habits. Previous health good. Four or five years ago had a vesicular eruption of the legs, attended with redness, great heat, itching, and a discharge of a watery-looking fluid. He states that he had at the same time erysipelas of the face. The present affection of the legs began between four and five months ago. It commenced, as the former, with an eruption of vesicles, accompanied with redness, heat, itching, and tension of the skin, first on the right, and soon after on the left leg.

*Present Symptoms.*—Both legs, from the feet nearly to the knees, are of a bright, deep red colour, glistening, and moist all over, from the continual oozing of a serous fluid from innumerable small abrasions of the cutis. Here and there, particularly at the margin of the redness, are seen the remains of a few vesicles, and also of bullæ, recognisable by the presence of circular portions of partially detached cuticle. There is a constant sensation of heat, itchiness, and tension, and great tenderness on pressure. There is no marked disturbance of the general health, but the pulse is hard and frequent; appetite good; bowels confined.

The diagnosis of this case is so conspicuous, from the description of the eruption, as to require from me no additional observations. Neither at the commencement, nor at the period at which we first saw it, could it have been taken for any other disease than eczema.

The irregular habits of this patient seemed to have predisposed him to attacks of the disease. It was the second time the legs were affected. Yet his general health was good. The treatment, therefore, has been nearly the same as in the preceding case, viz., at first antiphlogistic, and afterwards stimulating. Eight ounces of blood were taken from the arm, and the lead lotion was constantly applied, until the swelling, heat, tension, and itching subsided. We then employed the nitrate of silver lotion, six grains to an ounce of water, which was followed by a very considerable improvement. Hoping to effect a more speedy cure, the nitrate of silver was increased to fifteen grains, but this was too strong, for it was followed by tumefaction, the return of the heat and itching, and a discharge of serous fluid. We, therefore, laid aside the lotion, and have several times applied leeches to both legs, which have produced a highly beneficial change. We shall return again to the use of the lotion, but instead of applying it to the whole surface at once, we shall apply it, as in the former case, to small portions of each leg at a time.

We do not, I believe, possess any precise test for regulating the employment of the nitrate of silver in such cases. Generally speaking, it should not be employed until the heat and tension have been much subdued, and the redness has assumed a dull purple tinge, and more especially if the disease occupies an extensive surface. When the disease is limited to a small extent of surface, we may, even in the early and acute stage, employ this remedy freely and fearlessly, and with the greatest success.

There is one circumstance which may retard the cure in this case, viz., the thickening and induration of the subcutaneous cellular tissue, in several parts of both limbs, but particularly the right limb. Compression, by means of bandaging, will, probably, be the most efficacious means we can employ for their removal.

Having occupied the hour with these cases, I shall defer the consideration of the case of psoriasis guttata till another opportunity.—*London Lancet.*

---

*Formation of an artificial Anus, by opening the descending Colon without penetrating the Peritoneal Cavity.* By M. Amussat.—A lady, in Paris, æt. 48, long subject to obstinate constipation, sometimes preceded by hæmorrhage from the rectum, had vague pains in the pelvis and the lumbar region. Nothing, however, could be discovered further than a slight affection of the uterus, polypous excrescences of the urethra, and an old crural hernia on the right side.

She enjoyed good health for some time in the country, but on the 13th of May last returned to Paris, having suffered for eight days from obstinate constipation. On the 29th, M. Amussat was called in, the constipation still continuing. The rectum was found completely empty; but there was an obstacle above it, probably from a tumour in the pelvis, which no injections, nor any other mechanical means, could overcome. On the 2d of June, the constipation having existed twenty-four days, the tympanitis increasing, and the patient suffering intolerable pain, and earnestly requesting an operation, it was decided on, and performed with the assistance of MM. Breschet, Recamier, &c.

The patient being laid on her face, a transverse incision was made over a prominence which was distinctly visible on the left flank, two fingers' breadths above the crest of the ilium, and extending from the outer edge of the sacro-lumbalis to the middle of the crest of the ilium. The several layers of muscle and fascia were divided till the adipose tissue immediately covering the intestine was exposed; this was then cut through, and two threads were passed through the walls of the intestine, at each end of the wound, to retain it and prevent it from collapsing. Having clearly exposed the colon, of which a large space was uncovered by peritoneum, a fine trocar was plunged into its most prominent and distended part, and gases and fluid fæcal matter escaped through the canula. Immediate ease followed. A probe-pointed bistoury was passed in by the side of the canula; and when the aperture in the intestine was increased in both directions, gas and fæces flowed out in abundance. The patient expressed the greatest relief, and her countenance lost all its anxious expression, and its livid hue. The intestine was washed out with injections, and the edges of the opening in it were attached to those of the wound in the abdominal walls by four sutures.

The consequences of the operation were in no respect unfavourable. Sixteen days after it the patient was perfectly well, and had returned to her usual habits, and every thing induced her attendants to hope that, as far as the tumour in the pelvis, from which the intestinal obstruction arose, would permit, her recovery would be permanent.—*London Medical Gazette, from Comptes-Rendus, Juin* 17, 1839.

---

*Discovery of Muscles which rotate the Dorsal Vertebræ, (rotatores dorsi,) in Men and Mammalia.* By Prof. Theil, of Bern.—The rotatores dorsi are muscular fasciculi situated beneath the multifidus spinæ, and separated from its fasciculi by cellular tissue. They are found only in the dorsal portion of the spine, and are usually eleven in number on each side. They arise from the transverse processes of the dorsal vertebræ, from the second to the eleventh, and each is attached by fibres running transversely inwards, to the arch of the vertebræ next above that from which it arises. Each muscle, covered by the multifidus spinæ, is fixed by short tendinous fibres to the upper edge and posterior surface of the transverse process, and by fleshy fibres to the lower edge, and in part to the posterior surface of the arch, up to the base of the spinous process. The muscles have not all the same size; the lowest are largest, except the eleventh, which is small. The uppermost generally passes from the transverse process of the second dorsal vertebra, over the first, to the arch of the seventh cervical.

Professor Theil has discovered perfectly analogous muscles in the dorsal portions of the spine in several species of quadrumana, carnivora, and rodentia.—*Ibid., from Müller's Archiv.* Heft ii. 1839.

